# Balancing patient priorities for technical and interactional aspects of care in a measure of primary care quality

**DOI:** 10.1017/S1463423619000392

**Published:** 2019-06-26

**Authors:** Carol Mulder, Nadiya Sunderji

**Affiliations:** 1 Provincial Lead, Quality Improvement and Decision Support, Association of Family Health Teams of Ontario, Toronto, ON, Canada; 2 Lecturer, Department of Family Medicine, Queen’s University, Kingston, ON, Canada; 3 Psychiatrist-in-Chief, Waypoint Center for Mental Health Care, Penetanguishene, ON, Canada; 4 Assistant Professor, Department of Psychiatry, University of Toronto, Toronto, ON, Canada

**Keywords:** patient-centeredness, quality measurement in primary care, patient-provider relationship

## Abstract

**Aim:**

This study attempts to strike a balance to measure primary care quality in a way that considers what is important to patients, providers and the healthcare system, all at the same time.

**Background:**

The interest in delivering patient-centered primary care implies a need for patient-centered performance measurement. However, the distinction between measures of patient experience and technical aspects of care raises an unanswerable question: if a provider has good performance on technical measures but not on patient experience measures (or vice versa), what can be said about the quality of care?

**Methods:**

We surveyed patients to determine the relative priorities of each of a series of primary care measures in the patients’ relationship with their primary care provider. The on-line survey was co-designed with patient co-investigators. The items consisted of 14 primary care quality measures used in pre-existing performance report, 41 additional indicators including a novel set of patient-generated Key Performance Indicators and 17 questions about patients’ demographics, health and socioeconomic status as well as open-ended questions.

**Findings:**

Despite challenges, the study suggests that this is feasible. We argue that it is necessary to get better at measuring and finding ever-better ways to put patients at the center of primary care

## Introduction

“It is very nice to see anyone interested in asking patients what they would like to see from their physicians”.

In Canada, as elsewhere, there is widespread interest in ensuring that primary care is patient-centered. As the above patient comment indicates, patients are encouraged by this. The centrality of the patient–doctor relationship is one of the four principles of Family Medicine espoused by the College of Family Physicians of Canada (2006). In an era of increasing performance measurement and accountability, the interest in delivering patient-centered primary care implies a need for patient-centered performance measurement. In addition to the introduction of patient-reported outcome and experience measures (PROMs and PREMs, respectively) in public reporting of healthcare results, various tools have been developed to measure patient perceptions of primary care (Pearson and Raeke 2000; Stewart *et al.*, 2004; Doig *et al.*, 2015). Shi *et al.* (2001) developed a “Primary Care Assessment Tool” based on Starfield’s (1994) observation that quality in primary care is largely a factor of the relationship between patients and their providers. According to Starfield *et al.* (2005), the most important components of this relationship include comprehensiveness, continuity, coordination, and first-contact access.

However, the wider dialogue about the relationship between patient experience and quality underlines the persistent perception that these are separate constructs. Patient experience is rarely included as a domain in many quality frameworks (De Silva and Bamber, 2014). This is opposite to Starfield’s (1994) observations about the centrality of the relationship with patients in understanding the quality in primary care and the work of the Institute of Medicine which situates patient centredness as one domain of quality occurring in parallel with others such as effectiveness of care (IOM, 2002). The distinction between measures of patient experience and technical aspects of care when measuring quality perpetrates a competition for attention and accountability between these two perspectives in terms of assessing quality, such as that observed by Glenngård (2013). It asks an unanswerable question: if a provider has good performance on technical measures but not on patient experience measures (or vice versa), what can be said about the quality of care?

Part of the reason for the persistent dichotomy between patient experience and technical measures of quality relates to availability of data. Administrative data are readily available to track progress with technical aspects of care. In contrast, patient experience data are less readily available (Meltzer and Chung, 2014; Wong *et al.*, 2017) or highly variable (Fenton *et al.*, 2017). Data about patient-centeredness or the quality of patient–provider relationship tend to be even less widely available (Young *et al.*, 2017) or limited to one-time surveys as part of research projects such as QUALICOPC (as described by Li *et al.*, 2018) or, more recently in Canada, TRANSFORMATION (as described by Wong *et al.*, 2017). Others have attempted to incorporate a patient perspective in reporting by asking patients to prioritize technical and other measures of performance (Boivin *et al.*, 2014; Ivers *et al.*, 2014). The resulting priorities, while useful, do not reflect anything about the patient–provider relationship.

Notwithstanding the perceived importance of the patient–provider relationship and the continuing gap in data to assess it, the expectation for performance monitoring in primary care persists. This leads to a continuing focus on the measures for which data are available and a continuing perception of quality based on technical aspects of care.

Choosing not to report on performance is not an option. Therefore, we have taken a different approach to address the absence of data describing the patient–provider relationship in measurement of performance in primary care. We have introduced a composite measure that considers technical performance weighted according to the importance of each technical measure to the relationship between patients and providers. The composite measure is not a substitute for individual technical measures. We recognize the value of reporting on these, in part to meet regulatory requirements and also because providers generally find it easier to take action on individual technical measures than on composite measures (Scholle *et al.*, 2008). Instead, the composite is intended to give providers an overall sense of performance adjusted for what patients consider to be important in their relationships. The goal is to incorporate patient experience into the measurement of quality, rather than as a separate measure apart from other measures of quality. This study describes the process of generating the “weights” for generating a composite measure based on common technical measures used to monitor performance in primary care in Ontario.

Specifically, we asked patients: How important are [these commonly used primary care performance measures] to your relationship with your primary care provider?

## Methodology

### Approach

The Association of Family Health Teams of Ontario (AFHTO) is a collection of nearly 200 interprofessional primary care organizations, referred in Ontario, Canada, as teams and elsewhere as Patient-Centered Medical Homes. As a part of its broader efforts to demonstrate the value of interdisciplinary team-based primary care, AFHTO introduced a performance report called “Data to Decisions” (D2D). The D2D is a voluntary performance measurement initiative among primary care teams in Ontario. It was launched in 2014 and, over eight iterations and four years, has achieved high and sustained voluntary participation of more than 60% of the teams who are part of AFHTO. This study was launched in service of the D2D report. As noted above, we surveyed patients to determine the relative priorities of each of a series of D2D measures in the patients’ relationship with their primary care provider. We derived weightings from these priorities that could be applied in the calculation of a composite measure of quality. In this report, we discuss patient-reported priorities among the 14 quality measures in the D2D composite measure of primary care quality.

### Survey instrument

The survey was co-designed with patient co-investigators. The items consisted of 14 primary care quality measures used in D2D and 41 additional indicators from among those used by Southey and Heydon (2014) and a novel set of Key Performance Indicators developed by patient co-investigators (Patients Canada, 2015). We considered ability to get an appointment on the same or next day and ability to get an appointment in a reasonable time as measures of “access”, percent of visits to patient’s own provider as a measure of “continuity” and readmissions as our attempt to reflect “coordination”. We did not include a measure of “comprehensiveness” per se. This was as close as we could get to reflecting Starfield’s 4Cs and also manage to get data from enough teams.

**Figure 1.**
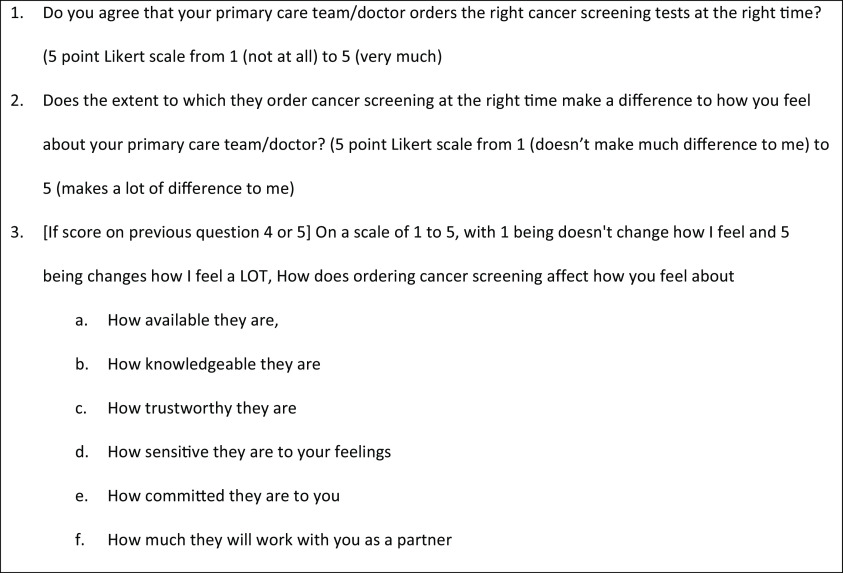
Example question from questionnaire

The question the survey was focussed on was: *if* your provider was excellent at [this quality measure], how would that affect your relationship with that provider? This is similar to a survey used to better understand partnerships between parents and pediatricians (Rapp and Pascoe, 2016). For example, patients might feel better about their relationship with their primary care provider if their provider had high performance on cancer screening indicators. If so, they would score “cancer screening” as “important to the patient provider relationship” (i.e., a score of 4 or 5 on a 5-point Likert scale). Patients were asked to evaluate each measure in this way. If a patient considered the measure to be important, they were then asked which aspects or domains of the relationship were affected by the concept. The domains (informed by the work of Southey and Heydon, 2014) were availability, knowledge, trustworthiness, sensitivity (to feelings), commitment to patient as a whole person and willingness to partner with the patient. The first 14 questions of the questionnaire related to the measures that were already part of the D2D composite measure of primary care quality. The questions for the remaining indicators were presented in two sections, and at the end of each section, patients were invited to continue with another set or conclude their participation if they wished. Figure 1 illustrates general pattern of the questions. The questionnaire also included 17 questions about patients’ demographics, health and socioeconomic status. Respondents were given an opportunity to comment on the process or content via two open-ended questions at the end of the survey. They were also invited to share input directly with the research team. The survey was available in English and French and was administered on-line via SurveyMonkey^TM^ (SurveyMonkey, n.d.).

The survey was piloted with colleagues and peers of the patient coinvestigators. The version reported here incorporated feedback from the pilot, and over 200 patients responded to the first iteration. A key change was the addition of a question distinguishing between perception of *performance* on an indicator and how *important* that indicator was in their relationship with their provider.

### Sampling and recruitment

The survey was conducted over three weeks in April 2017. Invitations to participate were sent via emails from Patients Canada and from primary care teams who were members of AFHTO, posts on AFHTO’s web page and the Twitter and Facebook presence of the patient co-investigators. All of the AFHTO’s, nearly 200 members, were invited to pass the message on to their patients. However, how many teams did so is not known. There were no explicit exclusion criteria. Response rates were not possible to estimate, given the unknown number of invitations sent and received.

### Data analyses

We generated descriptive statistics of respondents’ perceived priorities (question 2 in Figure 1) using IBM SPSS Statistics for Windows (2016), summarizing the proportion of respondents who “agreed” or “strongly agreed” that a measure was important to patients’ relationship with their providers.

## Results

The survey generated 218 responses. Slightly more than half of the respondents (55%) responded to invitations from primary care providers, with the remaining respondents recruited by contacting patient co-investigators. Sixty percent of respondents completed the first section of 14 items that are already used in the D2D composite measure, with lesser number of respondents for subsequent sections.

Patients appreciated the opportunity to be involved: “again I would like to thank you for considering patients’ input about the appropriate role that physicians should play in maintaining our health”. Nonetheless, they were clear that the questionnaire was confusing and frustrating: “I was initially very anxious to participate, but almost gave up in frustration halfway through” and “[I found it] unnecessarily restrictive and time consuming”.

Overall, the respondents were mostly healthy women of high socioeconomic status (Table 1).

**Table 1.** Demographics, health and socioeconomic status of respondents

Parameter	Number of respondents	Percent of respondents
Female	173	79.7
Male, unstated	44	20.3
Unstated age	12	5.5
0–18 years	1	0.5
19–34 years	38	17.5
35–49 years	58	26.7
50–64 years	75	34.6
65+ years	33	15.2
English-speaking preference	194	89.0
Other language preferences	24	11.0
Employment from income	136	62.3
Other sources of income (Benefits from Canada or Quebec Pension Plan, Child Tax Benefit or family allowances, Job-related retirement pensions, superannuation and annuities, Old Age Security and Guaranteed Income Supplement, Provincial or municipal social assistance or welfare, Registered Retirement Savings Plan/Registered Retirement Income Fund)	82	37.6
Annual income >$60,000	147	89.8
University-level education	189	69.3
Excellent or very good self-reported health	119	58.7
High level of social determinants of health (5 or 6 of the following supports: English speaking, income from employment, annual household income above $60,000 (CAN), someone to depend on, trust for advice and count on in emergencies)	143	88.3

Patients valued measures describing their interactions with providers more than measures relating to technical aspects of care. For example, three of the five highest priorities were related to interactional aspects of care: patients are involved in decisions about their care as much as they want to be, provider spends enough time with patients and [office staff are] courteous. Table 2 shows the first 14 measures in descending order of importance to patients’ relationships with their providers. The number of responses for the remaining measures was too low (*n* = 23) to generate meaningful conclusions so they are not discussed further in this paper.

**Table 2.** Patient priorities based on proportion of patients agreeing or strongly agreeing that the measure is important in their relationship with their provider

Measure: The extent to which the patient’s provider…	Respondents	Proportion of respondents
provides appointment in reasonable amount of time (ACCESS*)	151	0.89
involves you in decisions about your care	160	0.86
spends enough time	158	0.84
[office staff are] courteous	149	0.79
has access to ALL of your medical information	137	0.77
provides appointment on the same or next day (ACCESS*)	189	0.75
takes care of you at the office versus emergency department	145	0.74
makes it possible for you to see your OWN provider (CONTINUITY*)	141	0.73
sees you within 7 days of discharge from hospital (COORDINATION*)	135	0.7
gives children all the right vaccinations	133	0.68
orders the right cancer screening tests	183	0.66
screens you for diabetes and high blood pressure	156	0.65
has few patients who have to go to the Emergency Department	166	0.52
has few patients who have to be readmitted to hospital within 30 days of discharge (COORDINATION*)	135	0.47

* Element of Starfield’s 4Cs: first Contact (ACCESS), Continuity, Coordination, Comprehensiveness.

## Discussion

The respondents were predominantly female. However, for other parameters such as age distribution, health status and Emergency department utilization, the sample was similar to somewhat comparable data in the Canadian subset of the QUALICOPC survey (Li *et al.*, 2018).

This survey established relative priorities of various measures of primary care in terms of patients’ perceived relationship with their provider. Similar to the studies by Wensing *et al.* (1998) and Boivin *et al.* (2014), our findings showed that patients prioritize personal interactions higher than accessibility or technical performance in their relationships with providers. However, similar to the studies by Fung *et al.* (2005) and Cheraghi-Sohi *et al.* (2008), our study showed that the relationship with providers is also affected to some extent by performance on the technical aspects of care.

As planned, the results have been incorporated into a composite measure of quality that reflects the patients’ priorities regarding their relationships with their providers. The weights for each indicator in the composite may change with additional information from a more diverse sample than that are used here. Nevertheless, the results of this study demonstrate a way to build patient-centeredness into existing sets of performance measures. This represents a novel approach to alleviate the tension between these often-competing aspects of care and equips us now to examine the relationship between this composite measure of quality (with its built-in patient perspectives) and outcomes such as healthcare system utilization and cost.

In the meantime, as we had hoped, the survey made a difference. Family Health Teams (FHTs) in Ontario (who comprise 25% of the primary care sector) now use a composite measure of quality that incorporates patient perspectives (AFHTO, 2017). This remains unique in Ontario and Canada, 4 years after the introduction of the composite measure based on this study. FHTs now can and do consider how important indicators are to patients when choosing a QI focus. For example, if a team has sub-optimal performance on two indicators, they debate about which one is most important to patients before deciding on a QI plan.

## Limitations

The questionnaire generated frustration among participants based on length and complexity. In spite of our best efforts to revise the questionnaire, we were unable to completely address the similar input from the first survey and pilot testing. One challenge was the large number of indicators in D2D for which we needed to create weightings informed by patient perspectives. In addition, we felt that it was important to situate these weightings within the context of the patient–provider relationship. As a result of the anticipated issues with questionnaire completion, we were able to mobilize additional support to convene focus groups as an alternative approach to eliciting patient perspectives. This survey asked a complicated question: *if* your provider was excellent at the aspects of care reflected in the various measures, how would that affect your relationship with that provider? This, combined with the overall questionnaire length, likely affected the number and the completeness of responses. It also likely effectively excluded people who could not read English or French beyond a Grade 10 level. As we have already undertaken, focus groups or other methods to allow richer discussion of the question and potential responses might be a better approach for future attempts to generate similar data, even though they are more resource-intensive.

The sample was fairly homogenous, likely due to the abovementioned issues with the survey instrument, as well as the recruitment methodology, which largely relied on our patient partners. As with other patient surveys such as QUALICOPC (Li *et al.*, 2018), the homogeneity limits the generalizability of the results but does not preclude interpretation of patterns within the data related to relative priorities. However, the homogeneity did preclude exploration of the relationships between patients’ priorities and patient characteristics such as health or socioeconomic status. Input from a more diverse sample would allow calculation of weights reflecting the relative importance of indicators between patients with different health or socioeconomic status.

## Conclusions

This study illustrates one way to incorporate patient perspectives into quantitative measures of quality as a way forward in expanding the focus of performance measurement in primary care to include the relationship between patients and their providers. In our experience, patients’ enthusiasm for this work to be done suggests that while there is a role to refine the method of eliciting patient-perceived priorities, there is also a need to use and respond to these types of data, finding ever-better ways to put patients at the center of primary care. One of our patient partners summed it up best: “*You can’t keep asking what matters to patients but not changing in response to that. If you want to say you care about me, you need to do something about it!”*.
